# Axial ligand substitution in a microperoxidase-derived peptide enables P450-like electrocatalysis

**DOI:** 10.1039/d6ra04712b

**Published:** 2026-08-11

**Authors:** Koji Nakano, Sae Uezato, Hikari Nagadome

**Affiliations:** a Department of Applied Chemistry, Faculty of Engineering, Kyushu University 744 Motooka, Nishi-ku Fukuoka 819-0395 Japan k.nakano.qq@adm.fukuoka-u.ac.jp

## Abstract

A histidine-to-cysteine substituted minimal haem peptide exhibited P450-like electrocatalysis under direct electrochemical control. The immobilised peptide catalysed substrate oxidation on a gold electrode, consistent with a peroxide-shunt-type mechanism.

## Abstract 2

Minimal chemical modification reprograms haem peptide function.

## Graphical abstract

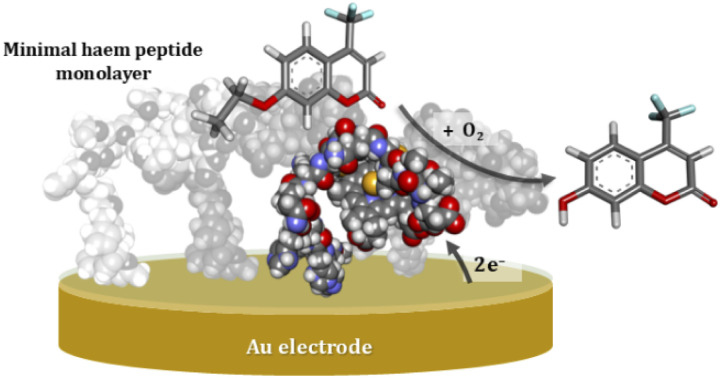

Haem enzymes catalyse a wide variety of reactions but share a common iron-porphyrin cofactor.^1^ Their catalytic properties primarily depend on the axial ligand; for instance, peroxidases utilise histidine, whereas cytochrome P450 enzymes (CYPs) utilise cysteine as the proximal axial ligand. While *de novo* protein engineering has evolved rapidly,^2,3^ an alternative approach involves extracting a minimal catalytic core from nature and reprogramming it *via* targeted modifications. Microperoxidases (MPs), which are fragments of the mitochondrial electron carrier cytochrome *c*, serve as minimal haem peroxidase models.^4^ Unlike most bulky haem enzymes, which often struggle with redox chemistry in electrochemical setups, these haem–peptide motifs support direct electron transfer (DET) due to their solvent-exposed haem. The undecapeptide MP11 has served as a platform for sequence and ligand modifications.^5,6^ We hypothesised that substituting the axial histidine with cysteine might enable CYP-like catalytic behaviour.

The heptadecapeptide, Ac-HHHHHHVQKCAQCCTVE-OH, was synthesised *via* microwave-assisted Fmoc SPPS. This sequence incorporates a specific histidine-to-cysteine substitution (H8C) relative to the MP11 sequence. The haem cofactor was subsequently reconstituted through a thiol–ene click reaction^7^ between the peptide and hemin, yielding the corresponding holo-heptadecapeptide, designated as MO17 (Fig. 1A). HPLC purification and MALDI-TOF MS analysis verified the identity of the synthesised product (Fig. S1 and S2).

**Fig. 1:**
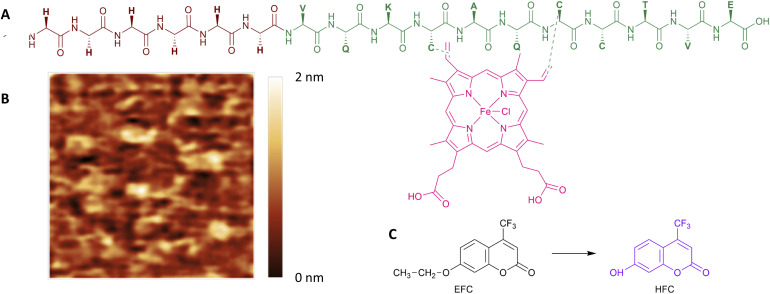
The proposed chemical structure of MO17 (A) and a close-up view of a captured frame obtained by liquid-phase HS-AFM imaging (Fig. S3), corresponding to a region measuring 200 × 200 nm. Panel (C) represents the oxidative *O*-deethylation of EFC by CYPs.

HS-AFM was employed to examine the morphology and dynamics of MO17 at the single-molecule level. An aliquot of aqueous MO17 solution (20 µM containing 0.1 M nickel(ii) acetate) was deposited onto a freshly cleaved mica substrate. HS-AFM imaging in the aqueous solution revealed dynamic, filamentous structures distributed across the mica surface (Fig. S3). As seen in Fig. 1B, close-up inspection of the captured frames revealed that these surface entities typically exhibit a contour length of approximately 30 nm in an extended conformation. Although this observed length exceeds the theoretical length of a 17-mer peptide, it remains within the expected range when accounting for the tip-sample convolution effect. These observations indicate that MO17 exists as a flexible molecule under the imaging conditions.

Next, FTIR spectroscopy confirmed the immobilisation of MO17 on the electrode *via* Ni(ii)-mediated immobilisation.^8^ An Au-coated glass substrate was immersed in an ethanolic solution of nitrilotriacetic acid self-assembled monolayer formation reagent (SNTA). After rinsing, the substrate was treated with an aqueous nickel(ii) acetate solution and then incubated with MO17. IRRAS measurements characterised the amide region of the MO17-treated SNTA self-assembled monolayers (SAMs) (Fig. S4). The resulting spectrum exhibited overlapping bands, which were qualitatively resolved into several components.^9^ Although precise peak assignments require further investigation, the spectrum clearly displays the amide I band at 1665 cm^−1^, the amide II band at 1529 cm^−1^, and an additional band at 1419 cm^−1^, the latter potentially arising from amide III vibrations and/or carboxylate stretches from the NTA SAM. Notably, the amide I frequency at 1665 cm^−1^ is characteristic of a random-coil conformation, which is highly consistent with the flexible structure observed *via* HS-AFM.

We measured MO17's electrochemical properties using a protein monolayer-modified Au electrode, prepared as described for the IR measurements. In the de-aerated electrolyte, only capacitive currents appeared on the NTA-modified electrode in CVs. After adding MO17, a distinct cathodic current was observed, indicating electrochemical activity (Fig. 2). These observations indicate that the peptide is immobilised in a form capable of exchanging electrons with the electrode. However, the cathodic current gradually decreased with repeated sweeps.

**Fig. 2:**
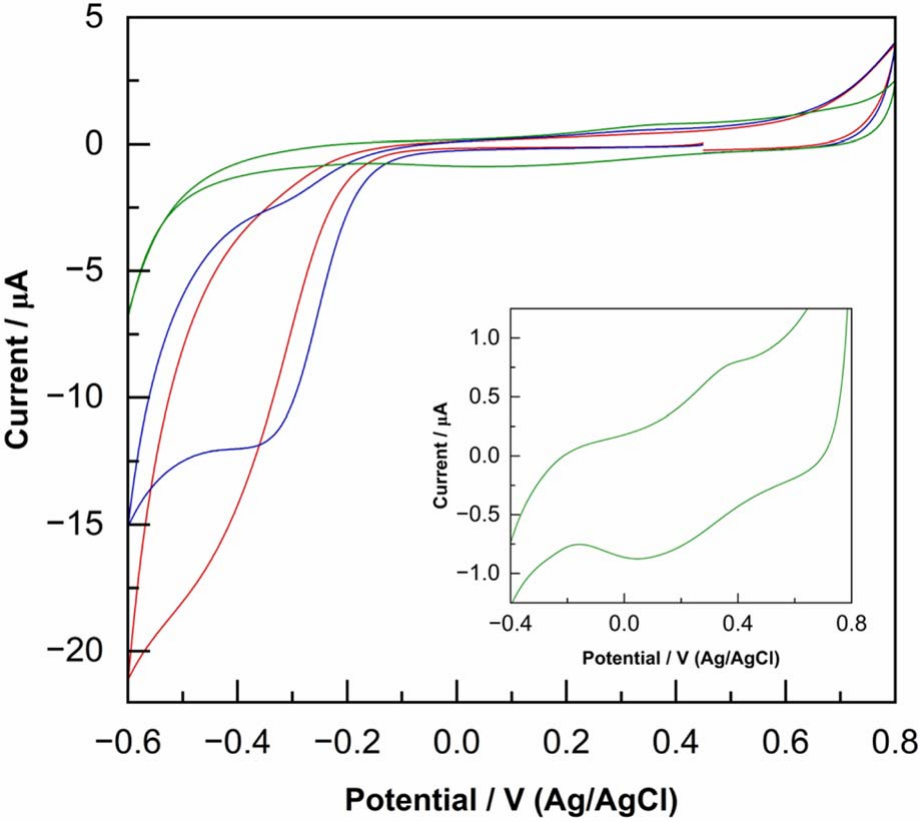
Representative CVs of the MO17-electrode obtained for the initial scan (blue) and the subsequent tenth scan (green) in deaerated (N_2_ gas) 10 mM phosphate buffer (pH 7) at a scan rate of 25 mV s^−1^ at 25 °C. For comparison, an initial CV for a different MO17-electrode under the same conditions but without N_2_ substitution is also shown (red). The inset is an expanded presentation of the tenth scan.

Natural and recombinant CYPs exhibit similar voltammetric behaviour when in an electrode-attached, DET-active state. Their cathodic currents, typically associated with electrocatalytic behaviour, have been attributed to the reduction of trace oxygen to hydrogen peroxide, which subsequently activates the CYPs *via* ferryl intermediates.^10^ This redox route effectively provides a peroxide shunt pathway (Scheme 1). The gradual decrease in current has been attributed to (i) the suppression of conformational changes necessary for the formation of the ferric–oxygen complex and (ii) partial denaturation of the CYP by hydrogen peroxide;^11^ we decided to consider this catalytic cycle as a plausible explanation rather than a directly demonstrated mechanism. For MO17, however, a subtle anodic peak at +0.344 V and a broad cathodic response at +0.121 V were observed after the catalytic current diminished. These features are consistent with redox processes involving ferryl haem species. Although further spectroscopic validation is required, this interpretation is consistent with the observed outcomes. Analysis of the peak separation^12^ yielded a heterogeneous electron-transfer rate constant (*k*_ET_) of approximately 0.05 s^−1^, suggesting that electron transfer is the rate-limiting step under the current conditions. The surface coverage (*Γ*), estimated from the oxidation charge, was approximately 13 pmol cm^−2^. This value lies between the reported values for cytochrome *c*^13^ and MP,^14,15^ which is consistent with the flexible, peptidic nature of MO17.

**Scheme 1:**
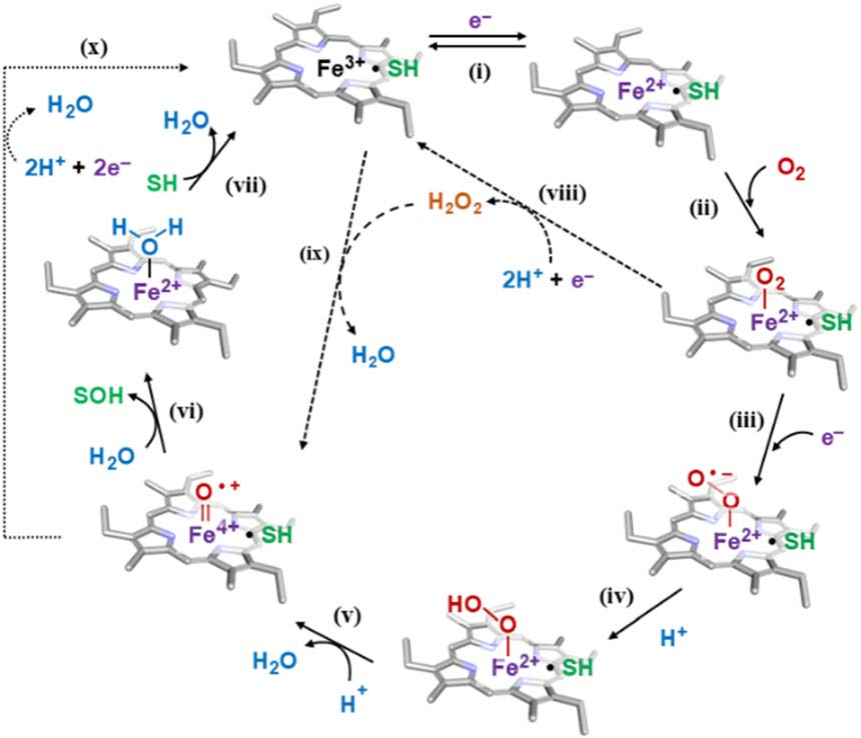
A CYP catalytic cycle adapted from previous reports^10,11^ with the indication of the peroxide shunt electrochemical activation pathway shown as the dashed lines. The electrochemical reduction pathway without the substrate molecules for the intermediary CYP-(Fe(iv) = O)˙+ is given as the dotted line.

The electroactive surface coverage estimated from the anodic charge under aerated conditions (9.0 pmol cm^−2^) was comparable to that obtained under nitrogen, indicating that the electrode preparation procedure yielded a similar amount of electrochemically addressable MO17 despite the different dissolved oxygen conditions.

Building on the characterisation of MO17, we evaluated its catalytic activity under electrochemical control by examining its voltammetric response toward a diagnostic substrate for CYP.^16^ CYP-mediated oxidative *O*-deethylation of 7-ethoxy-4-trifluoromethyl-coumarin (EFC) yields the fluorescent product, 7-hydroxy-4-trifluoromethyl-coumarin (HFC). To mitigate the effects of unavoidable oxygen-induced inactivation, a freshly prepared MO17-modified electrode was employed for each experiment, and data were collected from the initial potential sweep.

As shown in Fig. 3, the voltammetric response retained the characteristic current plateau observed in the absence of substrate; however, the current magnitude increased progressively with EFC concentration. This substrate-dependent enhancement was evident at sub-millimolar concentrations and culminated in a peak-shaped response at millimolar concentrations. Such behaviour is consistent with a regenerative electrocatalytic mechanism in which MO17 is reduced *via* DET, reacts with EFC, and is subsequently re-oxidised. At higher substrate concentrations, the interfacial electron transfer rate becomes limiting, preventing the attainment of a well-defined saturation current. Post-electrolysis HPLC analysis confirmed HFC formation, complemented by the detection of its characteristic fluorescence (Fig. S5 and S6). Although EFC is not selective for a single CYP isoform, formation of HFC is nevertheless consistent with P450-like oxidative chemistry.

**Fig. 3:**
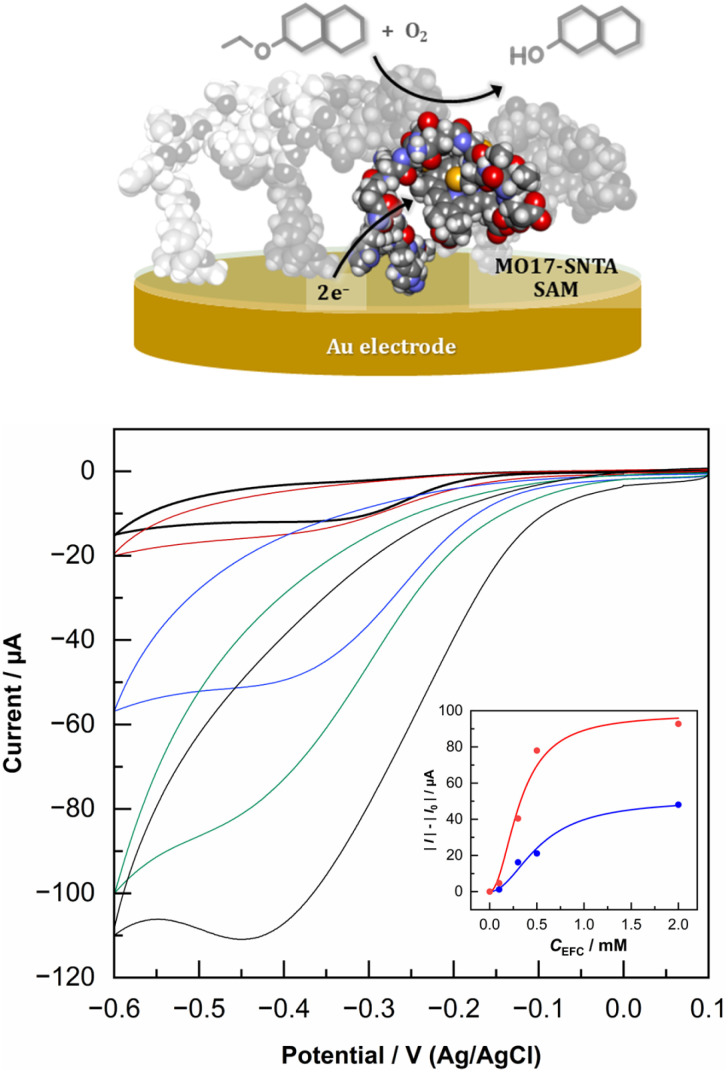
Summary of the CVs of the MO17-electrode in the presence of various concentrations of EFC: 0 M (bold black), 100 µM (red), 300 µM (blue), 500 µM (green), and 2 mM (thin black) (M = mol dm^−3^). Other measurement conditions were the same as those in Fig. 2. The inset shows the substrate-dependence of catalytic currents at −0.234 V (blue dots) and at −0.55 V (red dots) with the result of Hill-type fitting (blue and red curves). In the top, an expected DET pathway in the MO17-modified Au electrode is schematically shown.

Catalytic currents were obtained by subtracting the substrate-free background current recorded at the same potential (Fig. 3). Substrate dependence was evaluated at −0.234 V (near the wave midpoint) and at a plateau potential of −0.55 V *vs.* Ag/AgCl. Because only four substrate concentrations were accessible, the sigmoidal response was described empirically using a Hill-type relationship (eqn (1)), and the extracted parameters are reported as apparent values.1
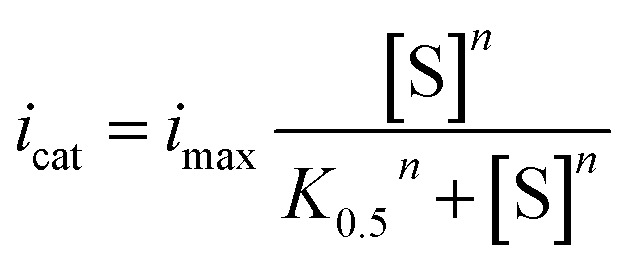


Residual analysis revealed systematic deviations for a Michaelis–Menten-type fit (Hill coefficient *n* = 1) at the plateau potential, particularly at low substrate concentrations, indicating that a simple hyperbolic model does not adequately capture the observed response (Fig. S7). In contrast, an empirical Hill-type fit with a fixed Hill coefficient (*n* = 2) substantially reduced these deviations (Fig. 3, inset and Fig. S7). At the midpoint potential (−0.234 V), differences between the two models were less pronounced, suggesting that interfacial electron-transfer effects partially mask the intrinsic substrate dependence.

On this basis, the apparent kinetic parameters derived at the plateau potential are considered more representative of the electrocatalytic behaviour of immobilised MO17; the apparent *V*_max_ and *K*_0.5_ thus obtained are 93 µA and 0.32 mM, respectively. Assuming a two-electron stoichiometry for the oxidative *O*-dealkylation of EFC, the apparent turnover frequency was estimated to be on the order of 40 s^−1^ (eqn (2); electrode area *A* = 0.50 cm^2^). It should be emphasised, however, that this value represents an apparent rate constant that reflects the combined contributions of electron transfer, chemical transformation, and possible peroxide shunt processes, rather than an intrinsic molecular *k*_cat_. This order-of-magnitude estimate highlights the catalytic potential of the minimal haem peptide scaffold under direct electrochemical control. Despite its minimal peptide framework, MO17 exhibits electrocatalytic substrate oxidation behaviour.

2 *i*_max_ = 2*FAΓk*_cat_

In summary, we show that axial-ligand substitution enables cytochrome P450-like electrocatalytic activity in a minimal haem–peptide system. The resulting system operates under direct electrochemical control, highlighting the catalytic potential of a simplified haem–peptide scaffold. While further spectroscopic studies will be required to establish the detailed axial coordination environment unambiguously, the available spectroscopic and electrochemical results, considered together with the molecular design, consistently support successful haem reconstitution and P450-like electrocatalytic behaviour. Accordingly, the proposed cysteine ligation should be regarded as an inference based on the combined structural design and experimental evidence rather than as a direct spectroscopic assignment. In addition, it cannot be ruled out that the H_2_O_2_ generated by the electrochemical process may be involved in the formation of HFCs. Further comparative studies using related haem–peptide systems together with additional surface characterisation and appropriate control experiments would be valuable for elucidating the detailed catalytic mechanism.

## Author contribution

K. N. conceived and designed the experiments. S. U. synthesized MO17 and also performed experiments, including RAIRS, and all electrochemical measurements. H. N. contributed by examining HS-AFM. K. N. analyzed the data and wrote the paper. All authors discussed the results and reviewed the paper.

## Conflicts of interest

There are no conflicts to declare.

## Supplementary Material

RA-OLF-D6RA04712B-s001

RA-OLF-D6RA04712B-s002

RA-OLF-D6RA04712B-s003

## Data Availability

The data supporting this article have been included as part of the supplementary information (SI). Supplementary information: synthetic procedures and analytical characterisation of the heptadecapeptide and MO17; RAIRS spectra; fluorescence and HPLC analyses of the electrocatalytic reaction products; kinetic fitting analyses of the voltammetric responses; and HS-AFM imaging data, including animated image files. See DOI: https://doi.org/10.1039/d6ra04712b.
