# Supplementary material for: Axial ligand substitution in a microperoxidase-derived peptide enables P450-like electrocatalysis
Source: RSC Adv. 2026 Aug 11;16(41):48095–8. doi: 10.1039/d6ra04712b (PMC13459061; doi:10.1039/d6ra04712b)
Supplement: Supplementary file 1 [file RA-016-D6RA04712B-s003.pdf]

Supplementary Information (SI) for RSC Advances.
This journal is © The Royal Society of Chemistry 2026

Supplementary Information

Axial Ligand Substitution in a Microperoxidase-Derived Peptide Enables P450-Like Electrocatalysis

Koji Nakano, Sae Uezato, Hikari Nagadome
Department of Applied Chemistry, Faculty of Engineering, Kyushu University. 744 Motooka, Nishi-ku, Fukuoka 819-0395, Japan

Contents

1. ***Synthetic procedure and analysis data*** 2
    1.1 Chemicals 2
    1.2 Synthesis of heptadecapeptide 2
    1.3 Reconstitution of heptadecapeptide by thiol-ene click reaction 5
2. ***High-speed atomic force microscopy (HS-AFM)*** 7
3. ***Reflection Absorption Infrared spectroscopy (RAIRS)*** 8
4. ***Analysis of the electrocatalytic reaction product of 7-ethoxy-4-trifluoromethyl-coumarin (EFC)*** 9
5. ***Voltametric behaviour of EFC and kinetic analysis*** 12

1. *Synthetic procedure and analysis data*

1.1 Chemicals

Rink Amide AM resin (200-400 mesh, 0.62 mmol g^{-1} loading) was purchased from Merck Millipore (Darmstadt, Germany). Fmoc-protected amino acids (Fmoc-AA-OH) were obtained from Watanabe Chemical Industries, Ltd (Hiroshima, Japan). Similarly, piperidine, o- (1H-benzotriazol-1-yl)-N,N,N',N'-tetramethyluronium hexafluorophosphate (HBTU), N-hydroxybenzotriazole (HOBt), and diisopropylethylamine (DIPEA) were supplied by the company. HPLC grade dimethyl formamide (DMF), dichloromethane (DCM), trifluoroacetic acid (TFA), acetic anhydride (Ac_2O), triisopropylsilane (TIS), and 1,2-ethanedithiol (EDT) were bought from Wako Pure Chemical Industries Ltd (Osaka, Japan).

1.2 Synthesis of heptadecapeptide

The heptadecapeptide, Ac-HHHHHHVQKCAQCCTVE-OH, was prepared by solid phase peptide synthesis (SPPS) at a 0.1-mmol scale in a manually operated microwave synthesis system (Discover® SP, CEM Co, Matthews, NC, USA). The corresponding amount of Rink Amide AM resin (162 mg) was settled in a reaction vessel and was swollen in 7 mL DMF overnight before use. For fmoc deprotection, 0.1 M HOBt in a 20%-piperidine/DMF solution was used. The activator base solution was 5%-DIPEA in DMF. The cleavage cocktail was prepared by mixing TFA (4.7 mL), TIS (0.05 mL), EDT (0.125 mL), and deionised water (0.125 mL). The synthetic procedure is as follows.

1. Wash the resin with 5 mL DMF and drain the solvent by filtration under reduced pressure. This treatment was repeated three times.
2. Fmoc deprotection of solid support. Add the fmoc-deprotection solution (5 ml). Apply microwave (MW) at 20 W. The reaction temperature was set to 75 °C, and the reaction mixture was incubated for 3 min; the solution was then drained. Wash the resin with 5 mL DMF, swirl for 10 s, and drain the solvent. Repeat four times.
3. AA coupling with resin. Each 0.5 mmol amount of Fmoc-Glu(OtBu)-OH and HBTU was dissolved in a 4-mL portion of the activator base solution, which was then added to the resin. Apply MW (20 W, 75 °C, 5 min) and then drain the solvent.

Wash the resin with 5 mL DMF, swirl for 10 s, and drain the solvent. Repeat four times.

4. Acetyl capping. Add an 8 ml portion of 25% Ac_2O in DMF, stand for 5 min, and drain the solvent. Wash the resin with 7 mL DMF, swirl for 10 s, and drain the solvent. This procedure was repeated four times.
5. Peptide chain elongation. Each 0.5 mmol amount of Fmoc-AA-OH and HBTU was dissolved in 4 mL of the activator base solution. Repeat steps 2 and 4 for each successive amino acid in the sequence in the following order: Fmoc-Val-OH, Fmoc-Thr(tBu)-OH, Fmoc-Cys(Trt)-OH×2, Fmoc-Gln(Trt)-OH, Fmoc-Ala-OH, Fmoc-Cys(Trt)-OH, Fmoc-Lys(boc)-OH, Fmoc-Gln(Trt)-OH, Fmoc-Val-OH, and Fmoc-His(Trt)-OH×6. Kaiser test confirmed the final coupling reaction. Wash the resin with 7 mL DMF, swirl for 10 s, and drain the solvent. Repeat four times.
6. N-terminal acetyl capping. Add the fmoc-deprotection solution (5 ml). Apply MW at 20 W. The reaction temperature was set to 75 °C. The reaction mixture was allowed to react for 3 min, after which the solution was drained. Wash the resin with 5 mL DMF, swirl for 10 s, and drain the solvent. Repeat four times.
7. Peptide release and side chain deprotection. Add the cleavage cocktail (4 mL), swirl for 10 s, and apply MW (20 W, 38°C, 30 min). Transfer the cleaved peptide solution by filtration under reduced pressure. Wash the resin with 4 mL TFA, then separate and combine with the peptide solution obtained in the previous step. Prepare 4 clean 15-mL centrifuge tubes and place 10 mL of cold diethyl ether in each. Equal portions of the peptide solution were poured into tubes and allowed to precipitate for at least 30 min. Centrifuge the precipitated peptide for four successive times (120,000 rpm, 30 min) at 0 °C to isolate the crude product and then carefully decant the supernatant. Repeat four times. The crude peptide thus obtained was dried under reduced pressure overnight.
8. Precipitation separation. Dissolve the peptide in 15 mL of deionized water. Freeze the sample with liquid nitrogen and lyophilize it overnight. The crude product was obtained as a white precipitate in glacial ether.
9. Peptide purification and analysis. HPLC examined the undecapeptide and was chromatographically purified on a C18-column (4.6 x 250 mm) if necessary (buffer A: 0.05% TFA in water; buffer B: 0.01% TFA in acetonitrile). HPLC elution (1

mL min^{-1}) was initialised at 85% buffer A that was linearly changed at time to 30% (0–30 min), 20% (30–35 min), 95% (35–45 min), and 95% (45–50 min). The fractions eluted at around 8 min were collected. Centrifugal condensation of the fraction followed by lyophilisation gave a pale yellow powder. MALDI TOFF-MS using α-cyano-4-hydroxycinnamic acid as matrix analysed the obtained products; *m/z* 2076.026 $[M+H]^+$ and for $C_{85}H_{126}N_{32}O_{24}S_3$ requires 2075.88.

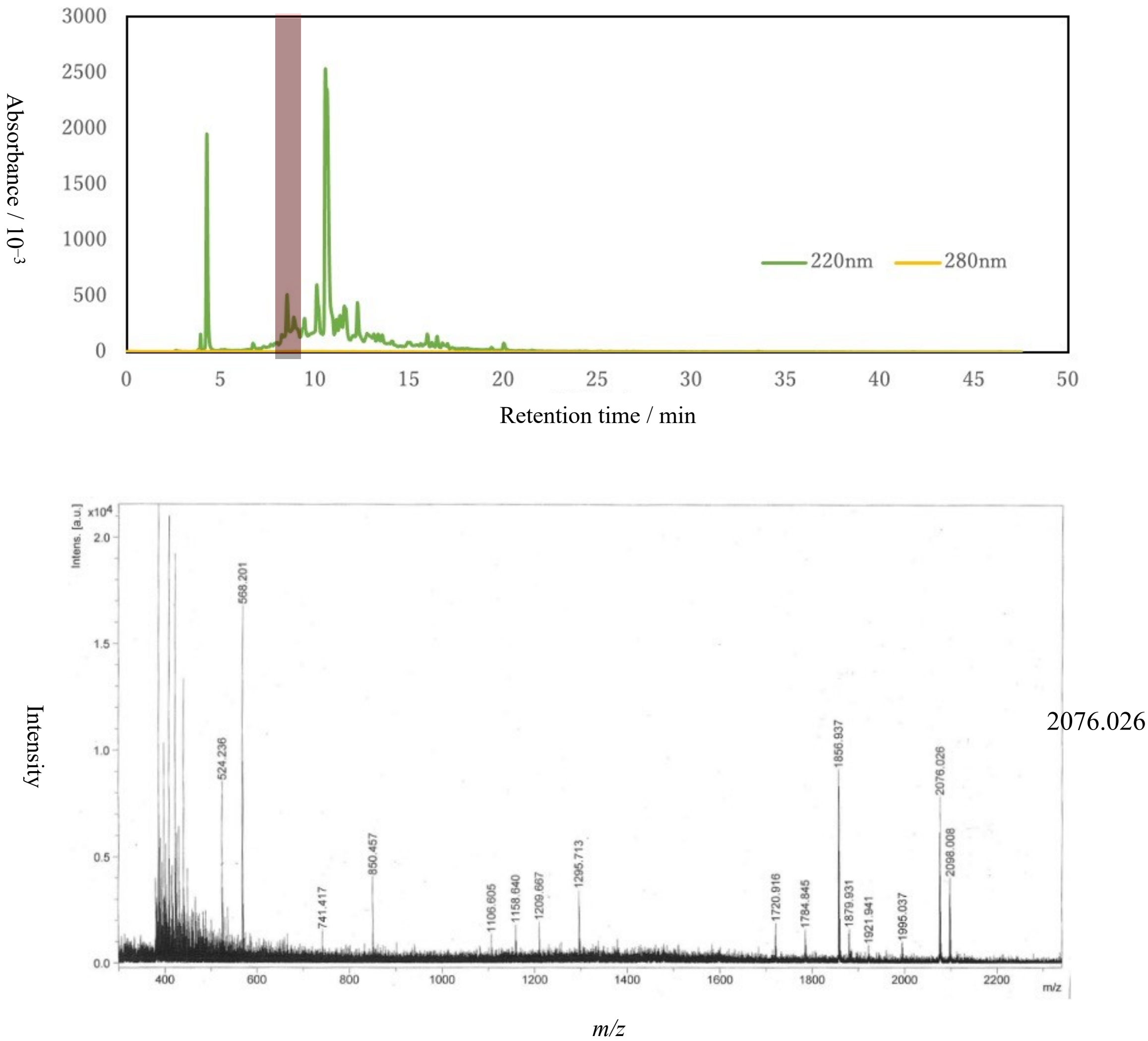

Fig. S1 Results of HPLC separation (top) and MALDI-TOF-MS analysis (bottom) for the heptadecapeptide.

1.3 Reconstitution of heptadecapeptide by thiol-ene click reaction

For the protein reconstitution experiments, hemin and 2-hydroxy-1-[4-(2-hydroethoxy)phenyl]-2-methylpropan-1-one (photoinitiator) were purchased from Tokyo Chemical Industry Co., Ltd. (Tokyo, Japan). Dithiothreitol (DTT) was supplied by Wako Pure Chemical Industries Ltd (Osaka, Japan). Other chemicals were reagent grade and used as received.

The heptadecapeptide was reconstituted with the prosthetic group by using the thiol-ene click reaction. A weighed amount of the heptadecapeptide (6.4 mg, 5.0 μmol) was dissolved in a 3.5-mL portion of aqueous 50%-THF solution that had been deoxygenated by slowly bubbling nitrogen gas. The solution was combined with 200-μL volume of aqueous 0.1 M NaOH containing 4.0 mg of hemin (6.0 μmol). Then, into the reaction mixture, was added one-third of a microspatulaful of the photo initiator DTT and sodium dithionite. The reaction was allowed to proceed by exposing to UV light (365 nm, 40 mW cm^{-2}) for 60 min at RT. The heptadecapeptide in the corresponding holo-structure was chromatographically purified on a C18 Column. The HPLC conditions were identical to those used for the heptadecapeptide. The fractions eluted from 12.5 min to 17.5 min were combined and centrifugally concentrated. The procedure resulted in a trace amount of reddish-brown powder after lyophilisation. As a typical case, we obtained a trace yield of 1.0% when 5 μmol of heptadecapeptide was subjected to the reaction, which was repeated 4 times, and the combined solution was HPLC-purified. MALDI-TOFF-MS using α-cyano-4-hydroxycinnamic acid as matrix analysed the obtained products, *m/z* 2725.417 for $[M+Na]^+$ for $C_{117}H_{154}ClFeN_{36}NaO_{28}S_3$ requires 2721.99.

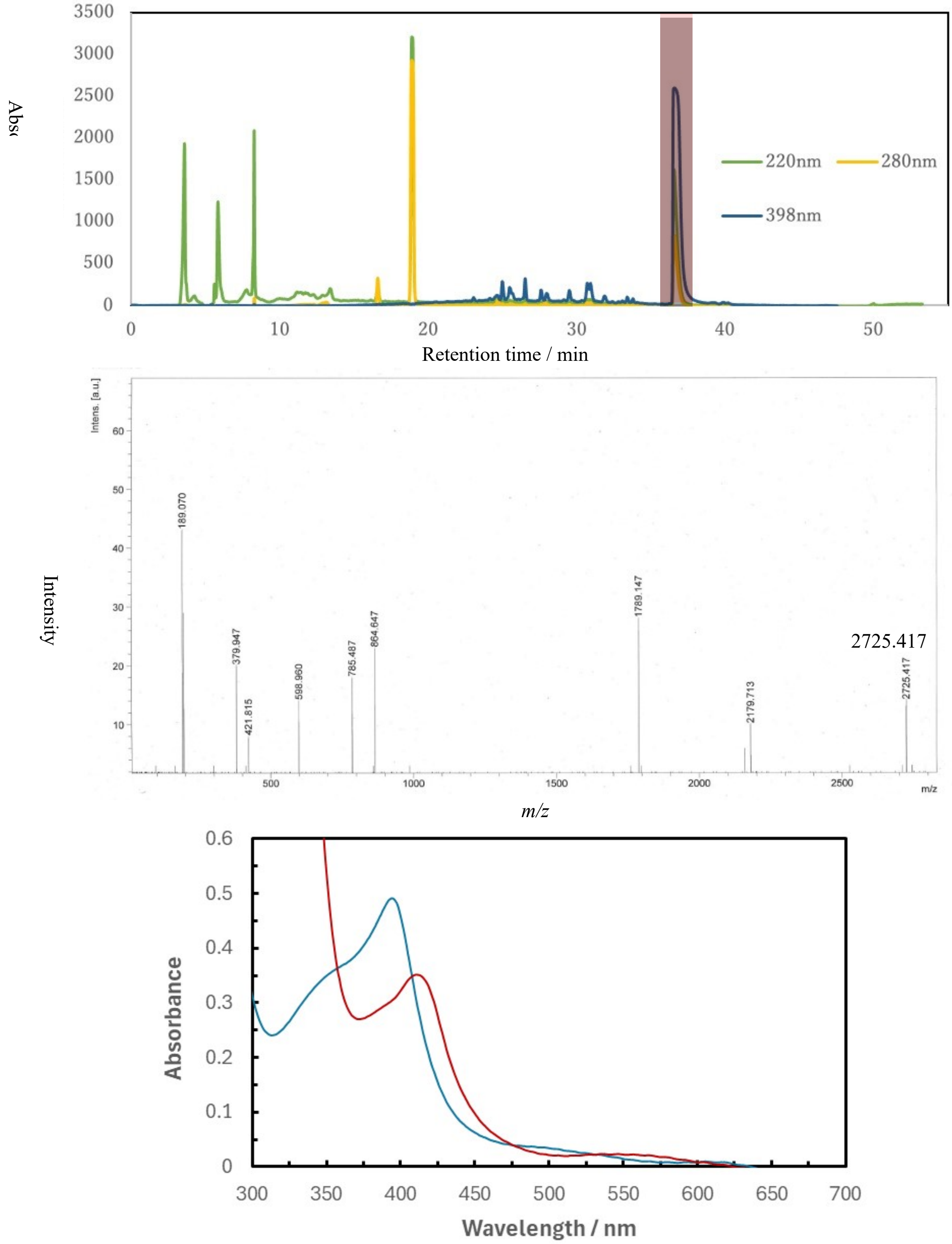

Fig. S2 Results of HPLC separation (top) and MALDI-TOF-MS analysis (middle) for the thiol-ene click reaction product of the heptadecapeptide. At the bottom are shown the UV–vis spectra of MO17 before (blue) and after (red) dithionite reduction, demonstrating the redox response of the reconstituted haem centre.

2. *High-speed atomic force microscopy (HS-AFM)*

HS-AFM imaging was made by using a High-Speed Atomic Force Microscope (Research Institute of Biomolecule Metrology Co., Ltd., Tsukuba, Japan). The cantilevers were typically silicon, with a tip radius of 10 nm, a length of 10 µm, a spring constant of 0.1 N m^{-1}, and a resonance frequency of 1,500 kHz. Freshly cleaved mica was used as the substrate, onto which a 5 µL aliquot of an aqueous solution of MO17 (20 µM) containing 0.1 M $Ni(CH_3COO)_2$ was deposited. The instrument was set up so that the droplet meniscus served as a fluid cell for the liquid-phase experiment.

Surface topological images were acquired continuously at 10 frames per second. All AFM data thus obtained were processed and analysed by using a copy of Gwyddion (David Nečas, Petr Klapetek, Gwyddion: an open-source software for SPM data analysis, *Cent. Eur. J. Phys.* **10** (1) (2012) 181–188). All experiments were made at room temperature, typically 22±2 °C.

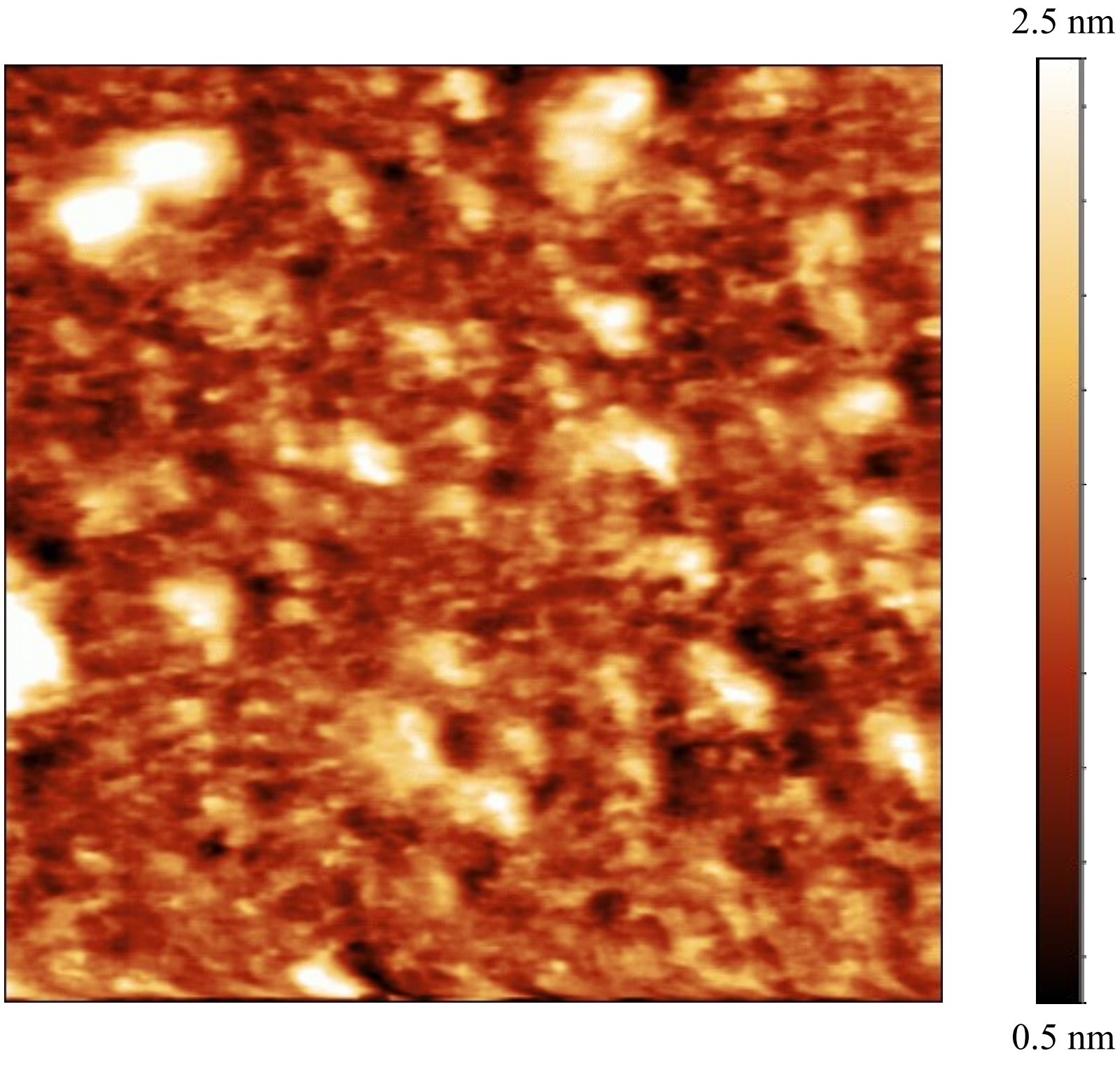

Fig. S3 Animated molecular images of MO17 obtained by liquid-phase HS-AFM, corresponding to a region measuring 800×800 nm.

3. Reflection Absorption Infrared spectroscopy (RAIRS)

Polycrystalline Au films, resistively deposited onto TEMPAX Float® glass plates (Kinoene Kogaku, Tokyo, Japan), were used as substrates. They were placed in the Plate Material Evaluating Cell (BAS Inc., Tokyo, Japan) and electrochemically etched by potential scanning from -0.4 V to +1.4 V in an aqueous 0.1 M H_2SO_4 solution containing 10 mM KCl. The cleaned substrate was subjected to self-assembly by immersing in a 10 mM ethanolic solution of nitrilotriacetic acid self-assembled monolayer formation reagent (SNTA, DOJINDO LABORATORIES, Japan) for 24 h, followed by Ni(II) chelation for 3 h using an aqueous 0.1 M $Ni(CH_3COO)_2$ solution. Finally, the substrate was treated with an aliquot of MO17 solution (100 µM in 50% aqueous trifluoroethanol) for 3 h. The substrate was rinsed with deionised water and dried under vacuum for 1 h.

IR spectra of the substrates were obtained using an FT-IR 620/V spectrometer (JASCO Co., Tokyo, Japan). The instrument was operated in the reflection–absorption mode using a polariser to eliminate s-polarised light and an incident angle of 80°. Spectral data were collected over 256 scans at 4 cm^{-1} resolution under vacuum.

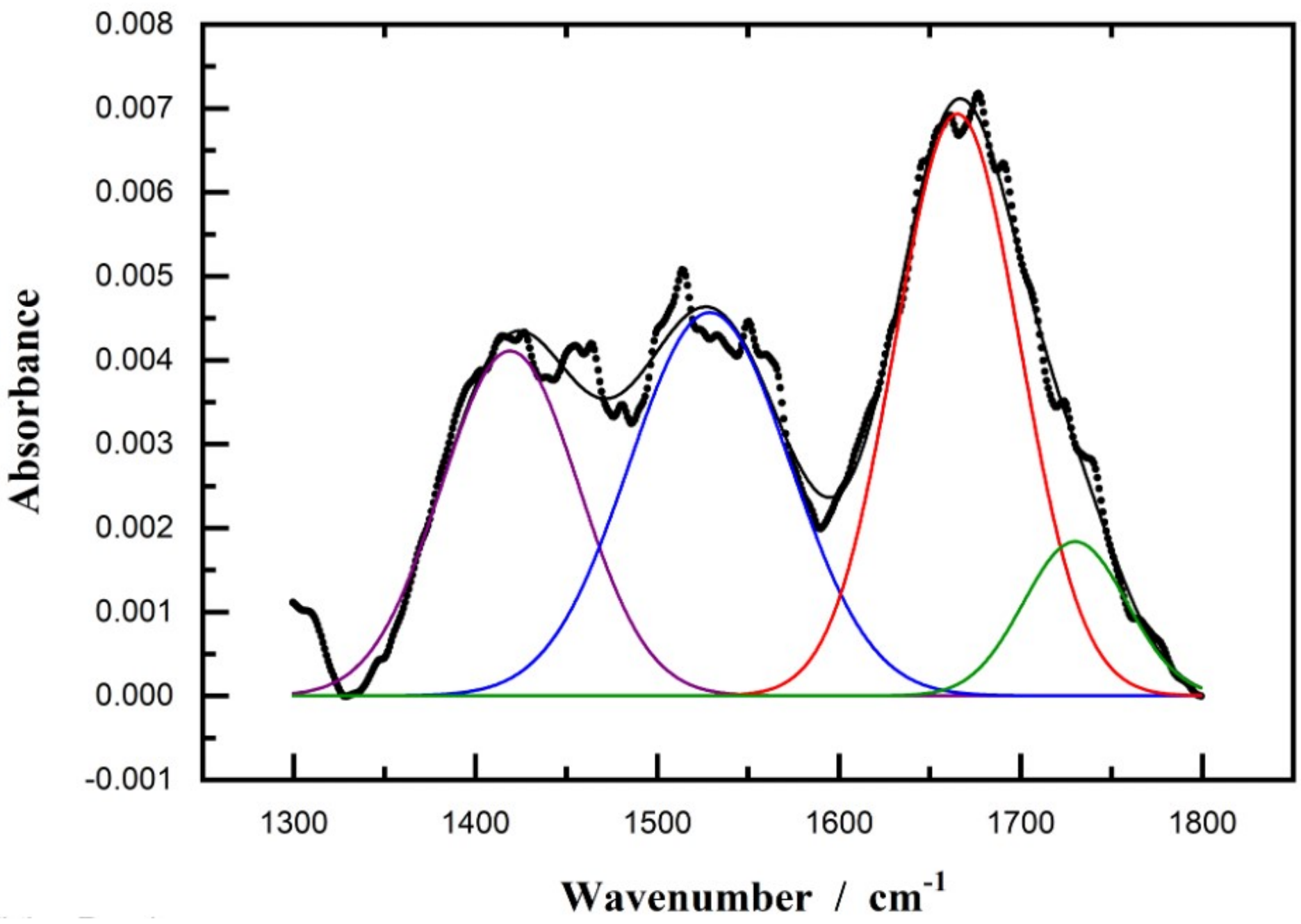

Fig. S4 A representative RA spectrum of an MO17-treated SNTA/Au substrate.

4. ***Analysis of the electrocatalytic reaction product of 7-ethoxy-4-trifluoromethyl-coumarin (EFC)***

To confirm the formation of the O-deethylation product, 7-hydroxy-4-trifluoromethylcoumarin (HFC), the reaction products generated at the MO17-modified electrode were analysed by fluorescence spectroscopy and HPLC.

First, fluorescence spectra of authentic HFC standard solutions were recorded using an excitation wavelength of 385 nm. Fluorescence spectra were then measured under identical conditions for aliquots of the electrolysed EFC solutions used for the experiments shown in Fig. 3 after tenfold dilution. The results are shown in Fig. S5.

The authentic HFC standard exhibited strong fluorescence emission centred at approximately 500 nm, consistent with the reported fluorescence properties of HFC. In contrast, the electrolysed samples showed a fluorescence maximum near 440 nm. Because EFC itself is known to exhibit weak fluorescence (excitation maximum 333 nm; emission maximum 415 nm), this signal was attributed primarily to residual unreacted EFC. Consequently, the background fluorescence from EFC made direct fluorescence quantification of HFC unreliable.

The reaction products were therefore analysed by HPLC with UV–vis detection. A 1 mL aliquot of 111 μM EFC in 10 mM phosphate buffer (pH 7.0) was electrolysed at an MO17-modified electrode by cyclic voltammetry between –0.5 and +0.5 V at a scan rate of 25 mV s^{-1}. After completion of the electrolysis, a 100 μL aliquot of the reaction solution was injected onto a C18 reversed-phase column and eluted using an acetonitrile/water mobile phase.

Chromatograms were recorded at 280 nm (common absorption wavelength of EFC and HFC), 330 nm (absorption maximum of EFC), and 385 nm (absorption maximum of HFC). UV–vis spectra of the individual chromatographic peaks were also obtained. As shown in Fig. S6, the peaks eluting at retention times of approximately 43 and 50 min were assigned to HFC and EFC, respectively, based on their retention behaviour together with their characteristic UV–vis absorption spectra.

To quantify product formation, a calibration curve was constructed using authentic HFC standards (Fig. S6F). From this calibration, the concentration of HFC in the electrolysed sample was determined to be 3.0 μM, corresponding to an EFC conversion of 2.7% under the present electrochemical conditions.

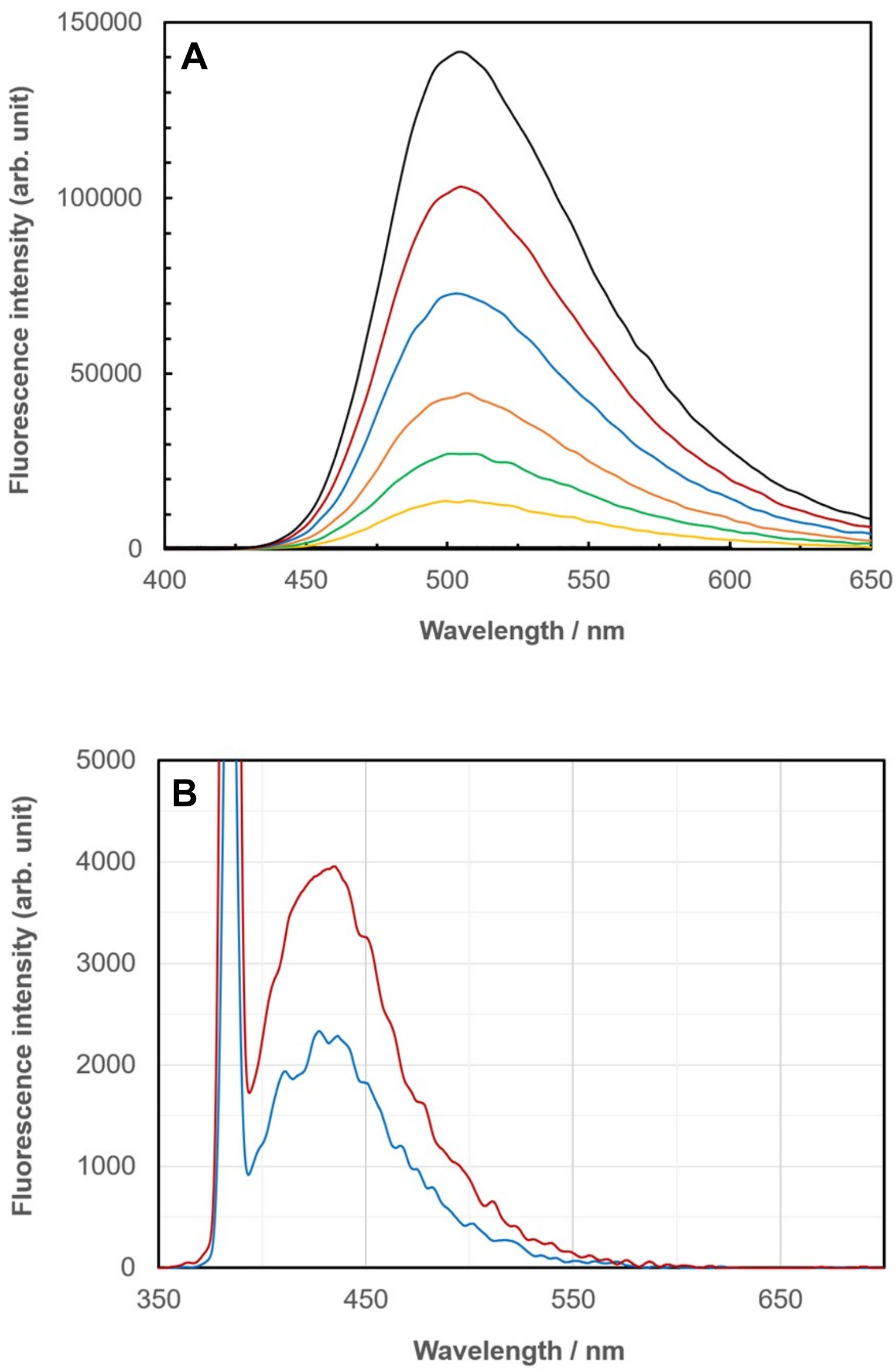

Fig. S5 (**A**) Fluorescence spectra for a series of HFC standard solutions of 0 μM (thick black), 1 μM (orange), 2 μM (green), 3 μM (brown), 5 μM (blue), 7 μM (red), and 10 μM (black). (**B**) Fluorescence spectra of the electrolytic solutions containing 500 μM (blue) and 2 mM (red) EFC after the cyclic voltammetric experiment shown in Fig. 3.

Absorbance at 280 nm

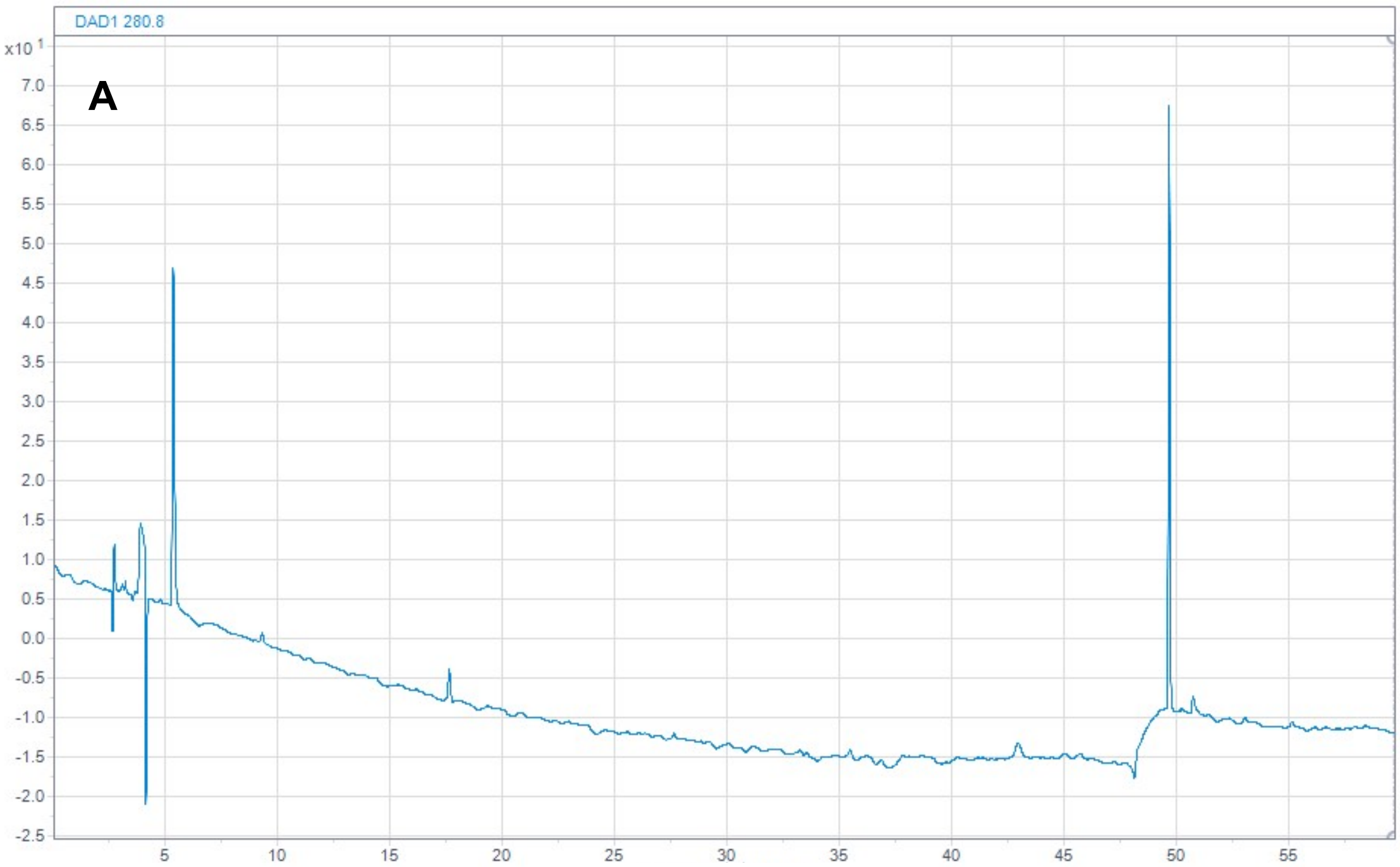

Retention time / min

Absorbance at 330 nm

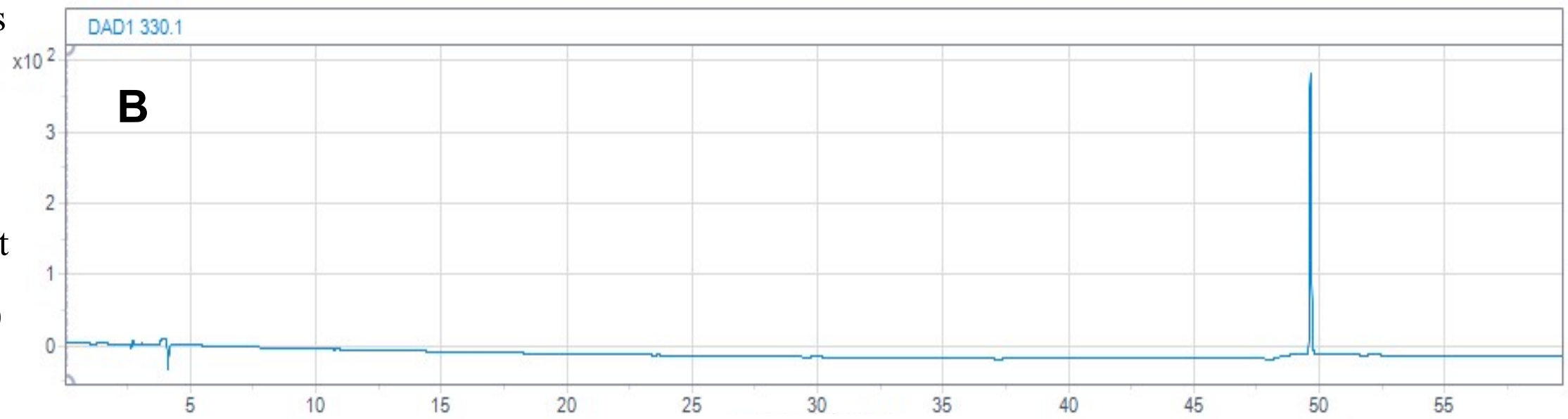

Retention time / min

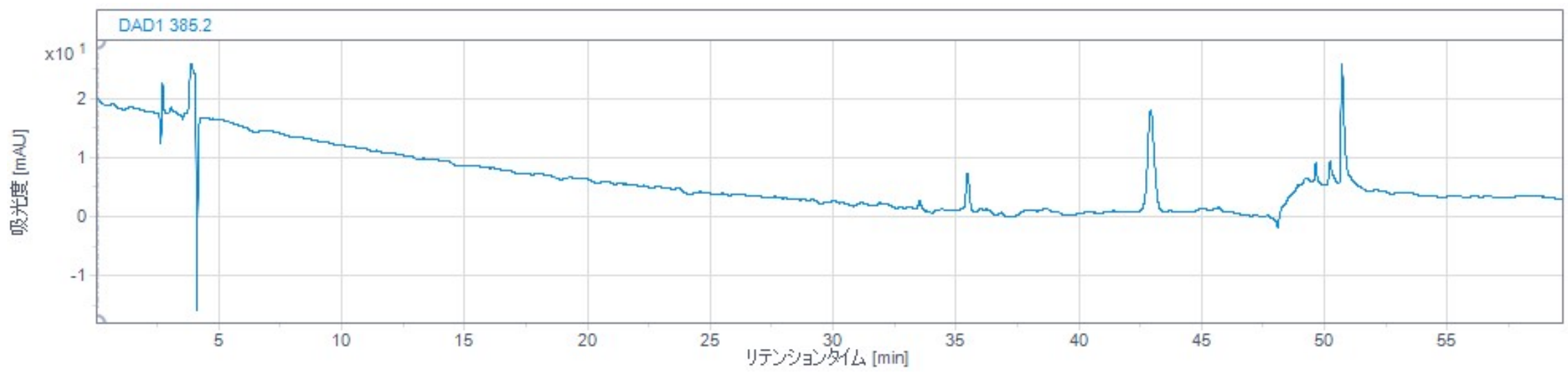

Fig. S6 (continued)

Absorbance at 385 nm

Retention time / min

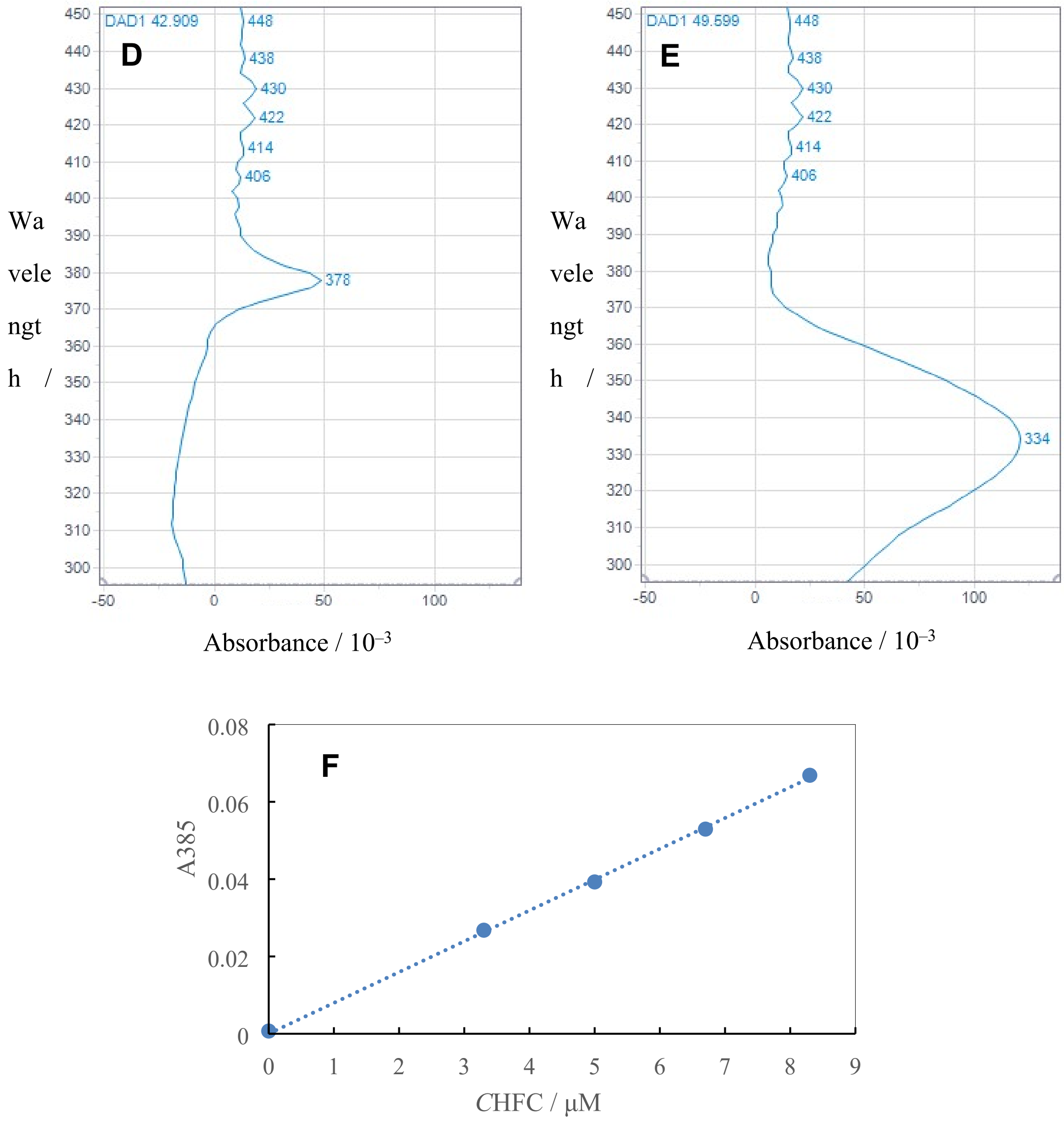

Fig. S6 HPLC charts of the electrolytic catalytic reaction product with UV-vis detection at 280 nm (**A**), 330 nm (**B**), and 385 nm (**C**). In panels (E) and (F), the UV-vis spectra found for the eluted component at each retention time of approximately 43 min (left) and50 min (right) are shown. At the bottom, the calibration graph for UV-vis determination of HFC (F) is shown.

5. *Voltametric behaviour toward EFC and kinetic analysis*

The MO17-modified electrode was prepared on the same Au-coated glass substrate using the identical immobilisation protocol employed for the RAIRS measurements. Substrate dependence of the catalytic currents was evaluated at −0.234 V (half-wave potential) and at a plateau potential of −0.55 V vs Ag/AgCl. Because only four substrate concentrations were available, the sigmoidal response was empirically described using a Hill-type relationship. Nonlinear curve fitting was performed using OriginPro 2022 (OriginLab Co., MA, USA). The extracted parameters are reported as apparent values.

Residual analysis (Figure S5) revealed systematic deviations for a Michaelis–Menten-type fit (Hill coefficient n = 1) at the plateau potential, particularly at low substrate concentrations, indicating that a simple hyperbolic model does not adequately capture the observed response.

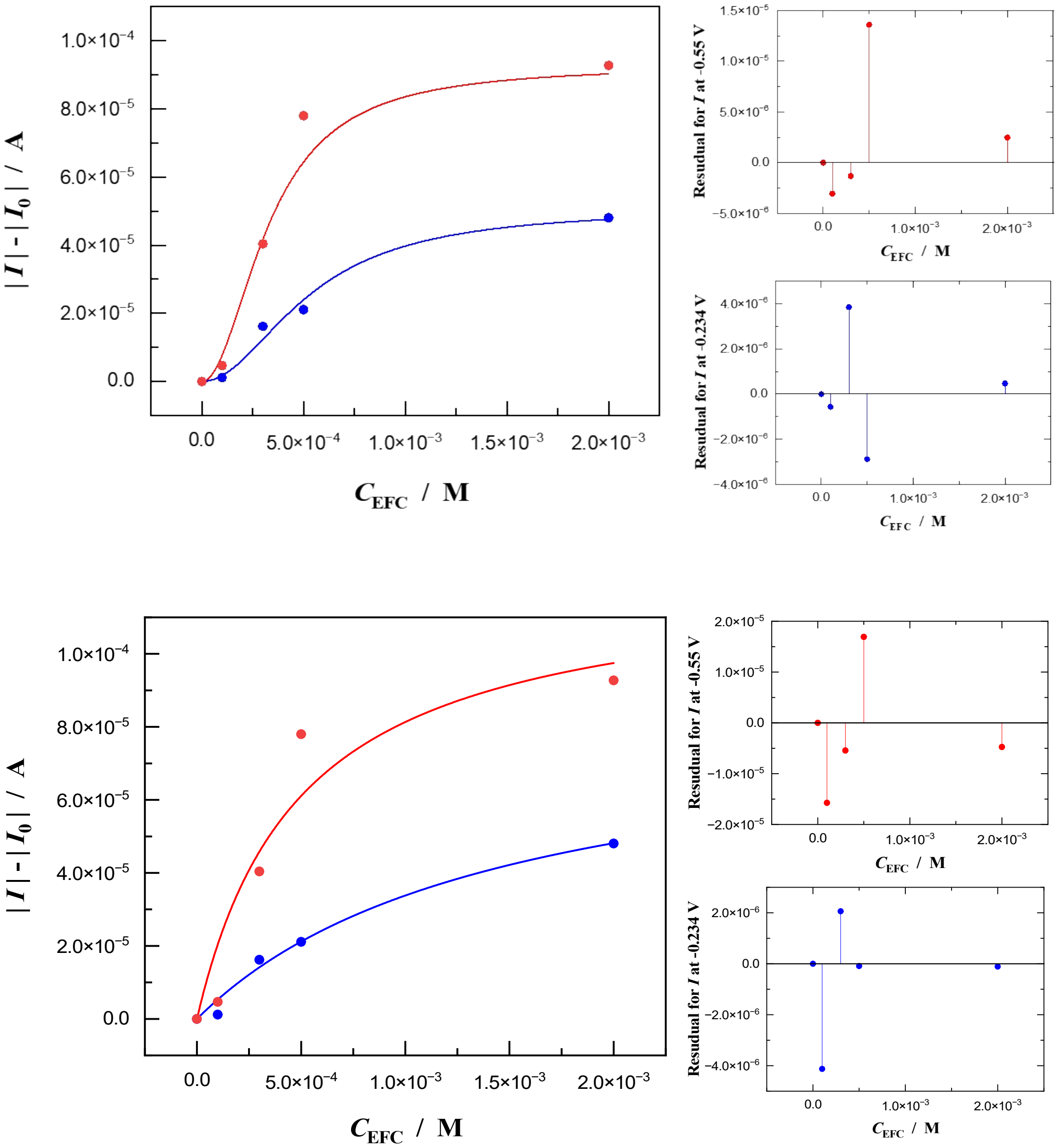

Fig. S7 Results of non-linear fits based on the Hill model (top) and the Michaelis–Menten model (bottom) for the EFC concentration dependence of the catalytic current of MO17-modified electrodes at -0.234 V (blue curve) and -0.55 V (red curve) in the left panel. The right panels are summaries of residual analyses for the corresponding data.
